# Supplementary material for: Psychosocial stress and neuroendocrine biomarker concentrations among women living with or without HIV
Source: PLoS One. 2021 Dec 23;16(12):e0261746. doi: 10.1371/journal.pone.0261746 (PMC8699620; doi:10.1371/journal.pone.0261746)
Supplement: Attachment — Submitted filename: PLOS ONE d-20-27314.edited.docx [file pone.0261746.s001.docx]

**“Psychosocial stress is associated with neuroendocrine biomarker concentrations**

**among Black women living with or without HI**" is a paper whose topic may be of interest to the readership of PLOS ONE. The current study takes an intersectional approach to examine the association between self-reported psychosocial stress and neuroendocrine biomarkers of stress among women living with HIV, with an emphasis on black women. This study contributes to the literature by focusing on understanding the potential mechanisms of cardiovascular disease among cis-gender women living with HIV, a population that has been neglected in research to date. This study supports and extends previous research by highlighting the role of stress, especially racial and gender-based harassment on the physiological functioning of black women living with HIV. However, I have one major concern about the misalignment between how the article is framed in the introduction and the results that are presented. What follows are my concerns and points of clarification.

Overall Concern

The authors frame the manuscript as an examination of stress in Black women living with HIV. This is present in the title and introduction. However, the authors actually examined the role of self-reported stress and biomarkers of stress among women of all ethnicities. As a result of this misalignment, the point of the paper gets lost. It appears the original research question was related to all women living with HIV as compared to those who were HIV-negative and that the sub-analysis among black women was part of a fishing expedition given the null findings of the main analysis.

Given the authors interest in understanding how race and gender-related stress impact health of women living with HIV, I recommend that the authors dedicate some time to fully addressing how racism and sexism may impact the stress and health of Black women living with HIV in the introduction; remove the analysis which included all women (currently the main analysis), and focus on the analysis examining the associations of interests among Black women living with HIV and those who were not. Alternatively, the authors might keep the analysis which includes all women as and include it with the other supplementary analysis that was conducted – however, I believe it would be best just to remove it.

Introduction

1. I would be careful when addressing how stress related to socioeconomic status is related to the health of women living with HIV. The authors do not measure socioeconomic status is a meaningful way (above or below $30,000 is not an approximate proxy for SES), therefore, the introduction should focus on the potential impact of racism, sexism, posttraumatic stress, and other perceived stress on the health of this population.

Methods

1. It should be noted that no traumatic event was indexed on the PTSD checklist. This is problematic because the “Stressful event” referenced in the questions may not meet criteria for a traumatic event. This is briefly noted in the limitations but should also be stated in the methods. The authors may also consider removing this measure all together since it is very difficult to differentiate between generalized anxiety and PTSD with understanding the index event. For example, the sample questions presented by the authors is “Avoiding activities or situations because they reminded you of a stressful experience”. It is difficult to distinguish between social anxiety and re-experiencing a traumatic event if there is no index event. Alternatively, the authors may separate the scores for racial and gender harassment and use them as separate indicators in the LCA. Therefore, they could still have three indicators of the LCA if they removed the PTSD scale.
2. Please clarify that the subgroup analysis conducted among Black women is stratified by HIV status.

Discussion

1. Again, the authors should be careful with attributing the stress of participants to SES, since SES was not appropriately measured and not included in the measurement of stress.
2. Also, the authors should be careful not to attributed PTSD symptoms to the experiences of violence reported by the participants, since no index event was reported for the checklist.

Decision: Revise and Resubmit, with Major Revisions
